# The predictive performance of SAPS 2 and SAPS 3 in an intermediate care unit for internal medicine at a German university transplant center; A retrospective analysis

**DOI:** 10.1371/journal.pone.0222164

**Published:** 2019-09-25

**Authors:** Michael Jahn, Jan Rekowski, Guido Gerken, Andreas Kribben, Ali Canbay, Antonios Katsounas

**Affiliations:** 1 Department of Nephrology, University Hospital Essen, University Duisburg-Essen, Essen, Germany; 2 Institute for Medical Informatics, Biometry and Epidemiology, University Hospital Essen, University Duisburg-Essen, Essen, Germany; 3 Department of Gastroenterology and Hepatology, University Hospital Essen, University Duisburg-Essen, Essen, Germany; 4 Department of Gastroenterology, Hepatology and Infectious Diseases, University Hospital Magdeburg, Otto-von-Guericke-University Magdeburg, Magdeburg, Germany; Azienda Ospedaliero Universitaria Careggi, ITALY

## Abstract

**Objective:**

To analyze and compare the performance of the Simplified-Acute-Physiology-Score (SAPS) 2 and SAPS 3 among intermediate care patients with internal disorders.

**Materials and methods:**

We conducted a retrospective single-center analysis in patients (n = 305) admitted to an intermediate-care-unit (ImCU) for internal medicine at the University Hospital Essen, Germany. We employed and compared the SAPS 2 vs. the SAPS 3 scoring system for the assessment of disease severity and prediction of mortality rates among patients admitted to the ImCU within an 18-month period. Both scores, which utilize parameters recorded at admission to the intensive-care-unit (ICU), represent the most widely applied scoring systems in European intensive care medicine. The area-under-the-receiver-operating-characteristic-curve (AUROC) was used to evaluate the SAPS 2 and SAPS 3 discrimination performance. Ultimately, standardized-mortality-ratios (SMRs) were calculated alongside their respective 95%-confidence-intervals (95% CI) in order to determine the observed-to-expected death ratio and calibration belt plots were generated to evaluate the SAPS 2 and SAPS 3 calibration performance.

**Results:**

Both scores provided acceptable discrimination performance, i.e., the AUROC was 0.71 (95% CI, 0.65–0.77) for SAPS 2 and 0.77 (95% CI, 0.72–0.82) for SAPS 3. Against the observed in-hospital mortality of 30.2%, SAPS 2 showed a weak performance with a predicted mortality of 17.4% and a SMR of 1.74 (95% CI, 1.38–2.09), especially in association with liver diseases and/or sepsis. SAPS 3 performed accurately, resulting in a predicted mortality of 29.9% and a SMR of 1.01 (95% CI, 0.8–1.21). Based on Calibration belt plots, SAPS 2 showed a poor calibration-performance especially in patients with low mortality risk (*P*<0.001), while SAPS 3 exhibited a highly accurate calibration performance (*P* = 0.906) across all risk levels.

**Conclusions:**

In our study, the SAPS 3 exhibited high accuracy in prediction of mortality in ImCU patients with internal disorders. In contrast, the SAPS 2 underestimated mortality particularly in patients with liver diseases and sepsis.

## Introduction

The use of in-hospital mortality prediction scores at admission to an intensive care unit (ICU) has become a viable evaluation method for treatment outcomes in critically ill patients [1]. To date, the Simplified Acute Physiology Score (SAPS) 2 and SAPS 3 are two of the most extensively validated risk prediction scores worldwide, and represent the most frequently applied scoring systems in European intensive care medicine [2–7]. In contrast to crude mortality data, such scores provide a risk-adjusted mortality assessment by considering various grades of disease severity and other prerequisites or predisposing conditions. Thus, standardized mortality ratios (SMRs) can be reliably calculated by comparing observed versus expected mortality rates [8]. Given an acceptable calibration performance, SMRs are useful tools, which can be used to evaluate interventions and/or quality of clinical management within an ICU or across a group of ICUs with comparable configurations over time. SMRs can also act as benchmarking parameters for performance assessment and improvement in an ICU with evolving cost-containment policies and medical practices or structures, as (for example) a decreasing SMR may indicate the presence of some particular change that is beneficially affecting mortality [9].

To date, in contrast to ICUs [2–4, 6, 7, 10–17], reports on routine application of mortality prediction scores in intermediate care units (ImCUs) are scarce [18–21]. Although such scores are considered important in decision-making processes in critical care, they are not yet standardly integrated in routine algorithms (e.g., via the hospital information system) in German ImCUs. Moreover, due to quite heterogeneous ImCU characteristics/settings, uniform validation of these scores remains challenging.

Meanwhile, significantly reduced in-hospital mortality rates have been reported for patients admitted to ICUs in hospitals with an ImCU relative to those admitted to ICUs in hospitals with no ImCUs [22]. These findings have added to the rising popularity of ImCUs in Germany, especially, for patients, which are too sick for the normal ward but also too stable to be admitted to the ICU [23]. The still relatively constrained ICU bed availability and the growing need to treat low/moderate-risk critical care patients in a more cost-effective manner have primarily driven this development. In the German hospital sector, ImCUs can be either integrated and/or adjacent to an ICU or run separately and independently from an ICU [24]. In fact, ImCUs may serve as “step-up” or “step-down” units between general wards and ICUs or as “subintensive” wards for patients coming from emergency or recovery rooms [25]. Although the majority of ImCUs treat surgical patients [24], the spectrum of medical support provided in each ImCU depends on the specific infrastructures and/or areas of expertise of the hosting clinics. However, continuous monitoring, non-invasive mechanical ventilation and application of vasoactive medications on demand represent common treatment modalities among ImCUs. Therefore, it is reasonable to further pursue evaluation of ICU scoring systems like the SAPS2 and/or the SAPS 3 in ImCU patients [24].

Taken together, this study aimed to analyze and compare performance of the SAPS 2 vs. the SAPS 3 in patients with internal disorders—who were admitted to an ImCU at a German university hospital´s transplant center—in order to improve the quality of local care evaluation and management. In addition, our data should be cross-interpreted with results from previous studies that evaluated the performance of both scores in non-surgical ImCU patients [18–21].

## Materials and methods

### Design, setting and patients

This study was conducted in a 22-bed ImCU for internal medicine at the University Hospital Essen, Germany, an academic clinical institution with a nearly 1300-bed capacity. This ImCU is headed by a team of gastroenterologists/hepatologists and nephrologists and runs independently from other intensive care units. Patients with acute or acute-on-chronic liver failure, acute kidney injury or chronic kidney disease (including patients requiring hemodialysis), patients receiving immunosuppressive therapy after solid organ transplantation (SOT; especially kidney and liver transplantation), and transplant candidates with various acute conditions accounted for the majority of ImCU admissions.

The nurse-to-patient ratio has been 1:4. The ImCU medical team involves 9 physicians (of those, 5 critical care specialists), i.e., 2 senior physicians and 7 residents, who work in 8- to 12-hour shifts. All beds are standardly equipped with continuous telemetry, pulse-oxymetry, body temperature as well as non-invasive and invasive monitoring of arterial blood pressure and central venous blood pressure. The bedside monitors are linked to a central unit that processes all relayed data and/or alarm signals.

Within a 18-month period, from January 2014 to June 2015, patients´ characteristics, medical history, reasons for admission as well as the worst clinical conditions and laboratory values were recorded (independently from medical interventions) within the first hour after ImCU-admission for SAPS 3 [26] or within the first 24 hours after ImCU-admission for SAPS 2 [27]. For both scores, predicted mortality rates have been calculated using the equations shown in Table 1. While SAPS 2 uses one general equation [27], customized formulas are available in different major geographic regions for SAPS 3; the present analysis has been carried out by using the SAPS 3 customized equation for North Europe [26].

**Table 1:** Simplified Acute Physiology Score (SAPS) 2 and SAPS 3.

SAPS 2: worst values for clinical and laboraty parameters within the past 24h after ICU-admission			SAPS 3: worst values for clinical and laboratory parameters within the first hour after ICU-admission		
**Clinical parameters**					
1.	Age [years]:	<40: 0p; 40–59: 7p; 60–69: 12p; 70–74: 15p; 75–79: 16p; ≥80: 18p	1.	Age [years]	<40: 0p; ≥40–60: 5p; ≥60-<70: 9p; ≥70-<75: 13p; ≥75-<80: 15p; ≥80: 18p
2.	Heart rate [bpm]:	<40: 11p; 40–69: 2p; 70–119: 0p; 120–159: 4p; ≥160: 7p;	2.	Heart rate, [bpm]	<120: 0p; ≥120-<160: 5p; ≥160: 7p
3.	SBP [mmHg]:	<70: 13p; 70–99: 5p; 100–199: 0p; ≥200: 2p	3.	SBP [mmHg]	<40: 11p; ≥40-<70: 8p; ≥70-<120: 3p; ≥120: 0p
4.	Body Temperature [°C]:	<39: 0p; ≥39: 3p	4.	Body Temperature [°C]	<35: 7p; ≥35: 0p
5.	Glasgow Coma Scale*:	<6: 26p; 6–8: 13p; 9–10: 7p; 11–13: 5p; 14–15: 0p	5.	Glasgow Coma Scale*	3–4: 15p; 5: 10p; 6: 7p; 7–12: 2p; ≥13: 0p
6.	Urine output [mL/24h]:	<0.5: 11p; 0.5–0.99: 4p; ≥1: 0p			
**Respiratory parameters**					
7.	If Mechanical Ventilation/CPAP or Pulmonary Artery Catheter in past 24h: PaO2/FiO2<100: 11p; PaO2/FiO2 100–199: 9 p; PaO2/FiO2≥200: 6 p		6.	If Mechanical Ventilation: paO2/FiO2<100: 11p; PaO2/FiO2 ≥100: 7p; No Mechanical Ventilation: PaO2 <60: 5p; PaO2 ≥60: 0p	
**Laboratory parameters**					
8.	Bicarbonate [mmol/L]:	<15: 6p; 15–19:3p; ≥20: 0p	7.	Creatinine, [mg/dL]	<1.2: 0p; ≥1.2-<2: 2p; ≥2-<3.5: 7p, ≥3.5: 8p
9.	BUN [mg/dL]:	<60: 0p; 60–179:6p; ≥180: 10p	8.	pH:	≤7.25:3p; >7.25: 0p
10.	Total Bilirubine [mg/dL]:	<4.0: 0p; 4.0–5.9: 4p; ≥6.0: 9p	9.	Total Bilirubine [mg/dL]	<2: 0p; ≥2-<6: 4p; ≥6: 5p
11.	WBC [x10^9^/L]:	<1.0: 12p; 1.0–19.9: 0p; ≥20: 3p	10.	WBC [x10^9^/L]	<15:0p; ≥15: 2p
12.	Potassium [mmol/L]:	<3: 3p; 3.0–3.9: 0p; ≥5: 3p	11.	Plateletes [G/L]	<20: 13p; ≥20-<50: 8p; ≥50-<100: 5p; ≥100: 0p
13.	Sodium [mmol/L]:	<125: 5p; 125–144: 0p; ≥145: 1p			
**Chronic diseases/Co-Morbidities**					
14. Metastatic Cancer: 9p;	15. Hematologic malignancy: 10p;	16. AIDS: 17p	12.	Cancer Therapy (e.g. Chemotherapy, Immunosuppression other, Radiotherapy, Steroid treatment): 3p; Metastatic Cancer: 11p; Haematological Cancer: 6p; Chronic Heart Failure (NYHA IV): 6p; Cirrhosis: 8p, AIDS:8p	
**Admission-related Parameters**					
17.	Type of admission:	Medical: 6p; Scheduled surgical: 0p, Unscheduled surgical: 8p	13.	ICU-admission	Planned: 16p; Unplanned: 19p
			14.	Surgical Status at ICU Admission	No surgery: 5p; Scheduled surgery: 0p; emergency surgery: 6p
			15.	Anatomical Site of Surgery	Transplantation surgery (Liver, Kidney, Panreas, Kidney and Pancreas, others): -11p Trauma (includes Thorax, Abdomen, limb or multiple): -8p; Cardiac surgery (CABG without valvular repair): -6p; Neurosurgery (Cerebrovascular accident): 5p; All others: 0p
			16.	Length of stay before ICU admussion [days]	<14: 0p; ≥14-<28: 6p; ≥28: 7p
			17.	Intra-hospital location before ICU admission	Emergency room: 5p; Other ICU: 7p; Other Ward: 8p
			18.	Vasoactive drugs before ICU admission	Yes: 3p; No: 0p
			19.	Reason(s) for ICU admission	Cardivascular: Rhythm disturbances (without simultaneous occurence of seizures): -5p; Hypovolemic hemorrhagic shock: 3 points, Hypovolemic non-hemorrhagic shock: 3p; Septic shock: 5p; Anaphylactic shock, mixed and undefinded shock: 5p; All others: 0p Hepatic: Liver failure: 6p; All others: 0p Digestive: Severe pancreatitis: 9p; Acute abdomen, Other: 3p; All others: 0p Neurologic: Intracranial mass effect: 10p; Focal neurologic deficit: 7p; Seizures: -4p; Coma, Stupor, Obtuned patient, Vigilance disturbance, Confusion, Agitation Delirium: 4p; All others: 0p
			20.	Acute infection at ICU admission	Nosocomial: 4p; Respitratory: 5p; All Others 0p
Logit = -7,7631+(0,0737*SAPS2)+(0,9971*ln(SAPS2+1)); Probability of Mortality: e^**logit**^**/(1+e**^**logit**^**)**			North European Logit: -26.9065+ln(SAPS3+5.5077)*6.; Probability of Mortality: e^**logit**^**/(1+e**^**logit**^**)**		

p = points; Glascow Coma Scale = GCS

*if patient is currently sedated, use estimated GCS prior to sedation

BUN = Blood Urea Nitrogen, WBC = White Blood Cell Count, SBP = Systolic Blood Pressure; bpm = beats per minute; pH = Hydrogen in concentration

During the study period, 73 out of 379 ImCU-admissions were identified as readmissions, and have therefore been withdrawn from further consideration. In addition, one patient had to be excluded due to incomplete data. Thus, data from 305 patients (all aged over 18 years) were subjected to final statistical analysis. In an effort to increase data reliability, we undertook quality control tests of data collected during a one-month pilot run, before launching the regular data acquisition phase.

The SAPS 2 includes 15 variables, i.e., 12 physiology variables, age, type of admission, and one variable related to underlying disease, which should be recorded in a 24-hour time window after admission [27]. The SAPS 3 utilizes 20 variables, i.e., 5 variables regarding patient characteristics prior to admission, 5 variables regarding the circumstances of the admission, and 10 physiology variables, which should be recorded within 1 hour before or after admission [26]. Table 1 provides a side-by-side overview of both scoring systems along with a detailed description of parameters included in each score. In-hospital mortality was the endpoint of this study.

### Statistical analysis

Statistical analysis was performed using SPSS (version 21.0; SPSS Inc, Chicago, IL) and SAS software (version 9.4; SAS Institute Inc., Cary, NC). For descriptive statistics, absolute and relative frequencies were calculated for categorical parameters, whereas continuous parameters were characterized using the median (MD) as well as the first and third quartile (Q1, Q3). For SAPS 2 and SAPS 3 mean and standard deviation (Mean ± SD) were also calculated. Inferential statistics to compare deceased with non-deceased patients included Fisher’s Exact Test for categorical variables and the Mann-Whitney U test for continuous variables. Results were considered statistically significant when *P*≤0.05.

**Fig 1:**
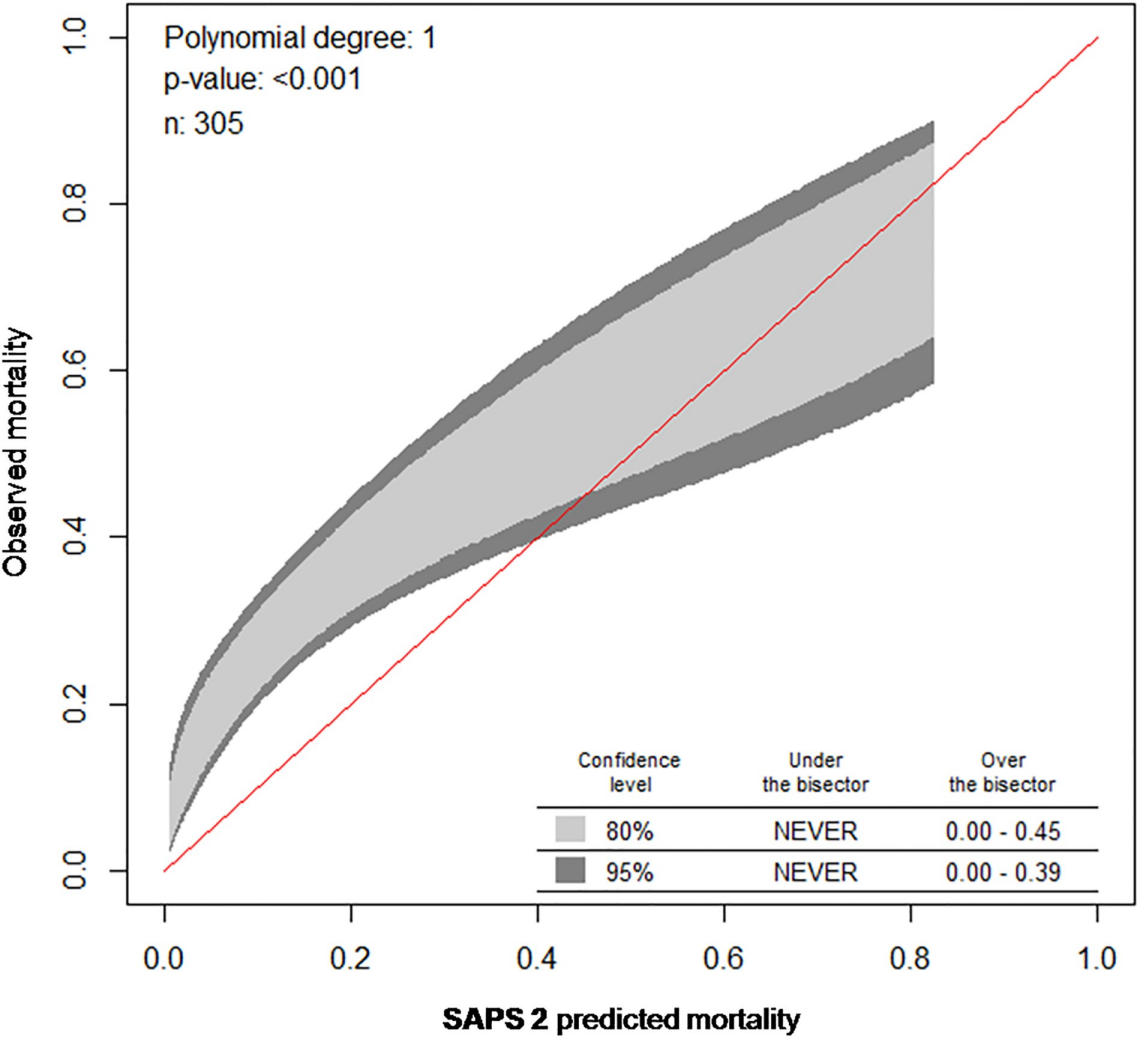
Calibration belt for the SAPS 2 model comparing observed and predicted ImCU mortality. Calibration curves with 80% (inner belt, light grey) and 95% confidence intervals (outer belt, dark grey) were created by plotting the predicted mortality (x-axis) against observed mortality (y-axis), thus if the curve is above the bisector (red line), it corresponds to an underestimation. The SAPS 2 calibration curve calibrates poorly where the bisector is not contained in the belt- this is the case in patients with low risk of mortality. SAPS 2 displays a poor calibration (*P*<0.0019).

**Fig 2:**
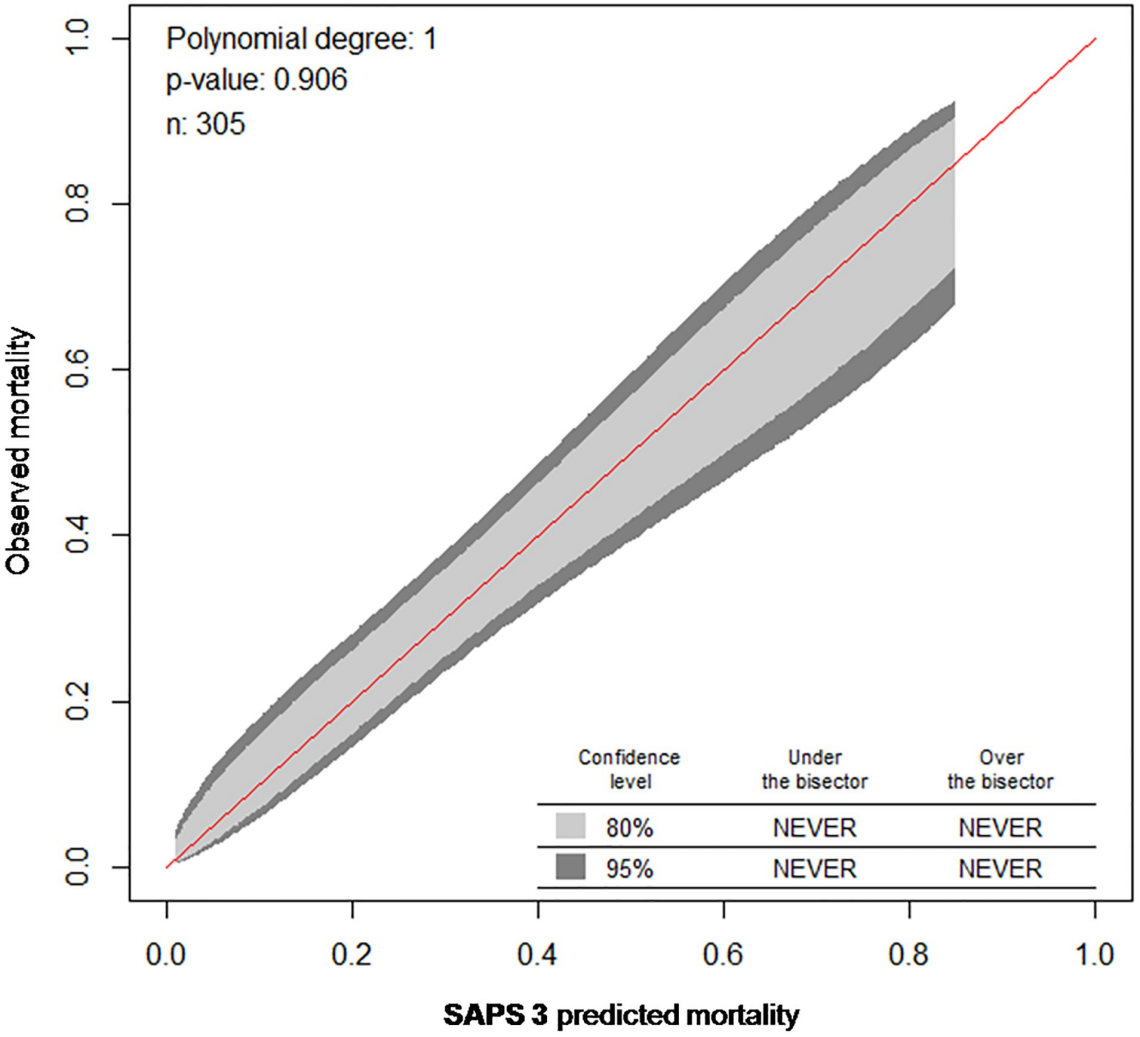
Calibration belt for the SAPS 3 model comparing observed and predicted ImCU mortality. Calibration curves with 80% (inner belt, light grey) and 95% confidence intervals (outer belt, dark grey) were created by plotting the predicted mortality (x-axis) against observed mortality (y-axis), thus if the curve is above the bisector (red line), it corresponds to an underestimation. Overall, the SAPS 3 calibrates well (*P* = 0.906).

The performance of prognostic models such as SAPS 2 and SAPS 3 encompasses two measures: discrimination and calibration. While the area under the receiver operating characteristic curve (AUROC) was calculated as measure for discrimination [28]), calibration was assessed using the Calibration belt [29, 30]. Note that a high p value (*P*>0.05) indicates a well-calibrated model. Calibration curves for the Calibration belt (Figs 1 & 2) were created by plotting the predicted mortality (x-axis) against observed mortality (y-axis). SMRs alongside their respective 95% confidence intervals (95% CIs) were determined for both SAPS 2 and SAPS 3 by dividing the observed mortality rate through the mean value of predicted mortality rates. Thus, we allow identifying over- (SMR < 1) or underestimation (SMR > 1) of the scores regarding mortality in our ImCU population.

### Ethics statement

This non-interventional study has been performed in accordance with the ethical principles and standards of the 1964 Helsinki declaration and its later amendments. The study protocol was approved by the local institutional review board of the University Hospital Essen (IRB: Ethik-Kommission am Universitätsklinikum Essen, 15-6436-BO). Because of the observational design of this cohort study, the institutional review board waived the requirement for patients’ informed consent.

## Results

### Patient characteristics

Of all patients (n = 305), one third (n = 92; 30.2%) was admitted via the department of gastroenterology and hepatology, and two thirds (n = 213; 69.8%) via the department of nephrology. Sources of patients’ admission were regular internal medicine wards at the University Hospital Essen or other hospitals (n = 226; 74.1%), the emergency department (n = 33; 10.8%), and the ICU (n = 45; 14.7%; Table 2). Median patient age was 60 years, the majority of patients were male (n = 184; 60.3%) and the median BMI was 25.7 kg/m^2^ (Table 3). The median stay in the ImCU was 6 days.

**Table 2:** Patient admission characteristics (n = 305).

(Intra)-hospital location before ImCU admission (SAPS 3)			
	regular care ward/ other hospital	226	74.1%
	emergency department	33	10.8%
	operating room	1	0.3%
	other ICU	45	14.7%
Type of admission (SAPS 2)			
	unplanned surgery	2	0.6%
	planned surgery	3	0.9%
	Medical	300	98.4%

**Table 3:** Patient characteristics at admission according to survival or death (n = 305).

Parameters	All Patients MD [Q1;Q3] or n, (range: min-max) or (% of all patients)	Survivals MD [Q1;Q3] or n, (range: min—max) or (% of Survivals)	Deaths MD [Q1;Q3], (range: min—max) or (% of Deaths)	*P* Significance
Patients	305	213	92	NA
Age (y)	60 [49;72] (range: 18–90)	58 [45;69], (range: 18–89)	63 [54;73], (range: 21–90)	0.0033*
Sex, n(%)	♂ 184(60.3%)	♂128 (60.1%)	♂ 56 (60.8%)	1.0000°
	♀ 121(39.7%)	♀85 (39.9%)	♀ 36 (39.1%)	
Body Mass Index (kg/m^2^)	25.7[22.1;29.0](range: 13.6–70.3)	25.4 [21.8; 28.9], (range: 13.6–48.2)	25.7 [23.3; 29.3], (range: 13.6–70.3)	0.4880*
**Chronic diseases / Co-morbidities**				
CKD	131(43.0%)	86 (40.4%)	45 (48.9%)	0.2075°
Cirrhosis ^§^	68 (22.3%)	30 (14.2%)	38 (41.8%)	<0.0001°
Solid Organ Transplantation	55 (18.0%)	41 (19.3%)	14 (15.2%)	0.5164°
Cancer & Cancer Therapy^§^	15 (4.9%)	10 (4.7%)	5 (5.5%)	0.7768°
Chronic Heart Failure (NYHA IV) ^§^	10 (3.3%)	4 (1.9%)	6 (6.6%)	0.0711°
Hematological Cancer ^§^	6 (2.0%)	4 (1.9%)	2 (2.2%)	1.0000°
AIDS ^§^	4 (1.3%)	3 (1.4%)	1 (1.1%)	1.0000°
**Acute conditions**				
AKI	97 (31.8%)	57 (26.8%)	40 (43.5%)	0.0049°
Cardiac Arrhythmias ^§^	52 (17.1%)	35 (16.4%)	17 (18.7%)	0.6218°
Liver Failure ^§^	50 (16.5%)	19 (8.9%)	31 (34.1%)	<0.0001°
Sepsis	43 (14.1%)	16 (7.5%)	27 (29.4%)	<0.0001°
Nosocomial Infection ^§^	26 (8.5%)	16 (7.5%)	10 (10.9%)	0.3732°
Vigilance Disturbances ^§^	26 (8.5%)	17 (8.0%)	9 (9.9%)	0.6550°
Respiratory Insufficiency with NIV	17 (5.6%)	12 (5.8%)	5 (5.5%)	1.0000°
Septic Shock ^§^	15 (4.9%)	11(5.2%)	4(4.4%)	1.0000°
Digestive Illnesses (e.g. severe pancreatitis, acute abdomen) ^§^	11 (3.6%)	10 (4.7%)	1 (1.1%)	0.1832°
Gastrointestinal Bleedings	10 (3.3%)	9 (4.2%)	1 (1.1%)	0.2918°
Focal Neurological Deficits ^§^	3 (1.0%)	3 (1.4%)	0	0.5568°
**Therapeutic interventions at admission**				
Vasoactive Drugs ^§^	188 (61.6%)	133 (62.7%)	55 (59.8%)	0.7000°
Hemodialysis	70 (23.0%)	41 (19.3%)	29 (31.5%)	0.0257°
NIV	17 (5.6%)	12 (5.8%)	5 (5.5%)	1.0000°
Stay in the ImCU (d)	6[3;14], (range: 0–115)	6 [3; 14], (range: 0–115)	7 [3; 22], (range: 1–67)	0.1197*
SAPS 2	30 [22;39], (range: 6–69)	27 [19; 36], (range: 6–65)	37.5 [27.5; 47.0], (range: 13–69)	<0.0001*
SAPS 2 predicted mortality	10.6% [4.7;23.0], (range: 0.5–80.6)	7.9% [3.3; 18.1], (range: 0.5–76.9)	20.5% [8.4; 39.2], (range: 1.5–82.6)	<0.0001*
SAPS 3	55[46;64], (range: 24–90)	51 [44; 59], (range: 24–88)	63 [57; 71], (range: 39–90)	<0.0001*
SAPS 3 predicted mortality	26.0% [12.0;44.0], (range: 1.0–85.0)	19.0% [10.0; 34.0], (range: 1.0–83.0)	42.0% [30.0; 58.0], (range: 6.0–85.0)	<0.0001*

MD = median, Q1 = first quartile, Q3 = third quartile, n = count, CKD = chronic kidney disease, AKI = acute kidney injury, NIV = non-invasive ventilation

^§^ = part of the SAPS 2 or SAPS 3 scoring system

NA = not applicable

* = Mann-Whitney U test

° = Fisher’s exact test

Among all chronic diseases taken into account by the SAPS 2 or the SAPS 3, liver cirrhosis (n = 68; 22.3%) showed the highest frequency in our cohort. Further co-morbidities like cancer and cancer therapy (n = 15; 4.9%), chronic heart failure at stage NYHA IV (n = 10; 3.3%), hematological cancer (n = 6; 2.0%) or AIDS (n = 4; 1.3%) were markedly less common. Moreover, we observed a high rate of patients with chronic kidney diseases (CKD) based on the KDIGO-criteria [31] (n = 131; 43.0%, of those, n = 39 with end-stage CKD) and patients with systemic immunosuppression after solid organ transplantation (n = 55, 18.0%; of those, n = 22 post liver transplantation, n = 28 post kidney transplantation, and n = 5 post combined pancreas-kidney transplantation, Table 3).

Regarding the SAPS 3 framework, the most frequent acute conditions at admission were cardiac arrhythmias (n = 52; 17.1%), followed by acute or acute-on-chronic liver failure (n = 50, 16.5%), nosocomial infections (n = 26; 8.5%), vigilance disturbances, e.g. coma, stupor, obtunded patient, agitation, confusion, delirium (n = 26; 8.5%), septic shock (n = 15; 4.9%), gastrointestinal emergencies, e.g. severe pancreatitis, acute abdomen (n = 11; 3.6%), and various focal neurologic deficits (n = 3; 1.0%). We also registered high rates of acute kidney injury based on the KDIGO-criteria [32] (n = 97; 31.8%) or sepsis according to Sepsis-2 criteria [33] (n = 43; 14.1%), and (less frequently) respiratory insufficiency requiring non-invasive ventilation (n = 17; 5.6%) or gastrointestinal bleeding (n = 10; 3.3%, Table 3).

Over half of all patients had to be treated with vasoactive drugs (noradrenaline, dobutamine, adrenaline or hemopressin; n = 188, 61.6%) at admission, while approximately one fourth of all patients (n = 70; 23.0%) received renal replacement therapy in terms of either intermittent hemodialysis (n = 62; 20.4%) or continuous cyclic or ambulatory peritoneal dialysis (n = 8; 2.6%).

### Performance of SAPS 2 vs SAPS 3

The median score (MD [Q1; Q3]) was 30 [22; 39] for SAPS 2 and 55 [46; 64] for SAPS 3, resulting in a median predicted mortality of 10.6% [4.7%; 23.0%] and 26.0% [12.0%; 44.0%], respectively (Table 3). Corresponding mean values and standard deviations were 31.4 ± 12.9 for SAPS 2 and 55.4 ± 12.9 for SAPS 3, causing mean predicted mortalities of 17.4% ± 17.6 and 29.9% ± 21.1 (Table 4). The observed in-hospital mortality was 30.2% (n = 92 patients; of those, n = 56 were men and n = 36 women), resulting in an SMR of 1.74 (95% CI, 1.38–2.09) for SAPS 2 and 1.01 (95% CI, 0.8–1.21) for SAPS 3, respectively (Tables 4 and 5). Hence, while SAPS 2 heavily underestimated the true mortality rate, SAPS 3 provided a markedly accurate prediction.

**Table 4:** Performance of SAPS 2 and SAPS 3 scores in the ICU.

Scoring system	Score	Predicted mortality	SMR	Calibration belt	AUROC
	Mean± SD	Mean± SD	(95% CI)	*P* value	(95% CI)
SAPS 2	31.4 ± 12.9	17.4% ± 17.6	1.74 (1.38–2.09)	<0.001	0.71 (0.65–0.77)
SAPS 3	55.4 ± 12.9	29.9% ± 21.1	1.01 (0.8–1.21)	0.906	0.77 (0.72–0.82)

SMR = Standard Mortality Ratio, AUROC = Area Under the Receiver Operating characteristic Curve, SD = Standard deviation, CI = Confidence Interval

**Table 5:** Case specifications at ImCU-admission and SMRs based on SAPS 2 and SAPS 3.

Statistics	Total	Deaths	SAPS 2 Mean ± SD	SMR (95% CI)	SAPS 3 Mean ± SD	SMR (95% CI)
All	305	92 (30.2%)	31.4 ± 12.9	1.74 (1.38–2.09)	55.4 ± 12.9	1.01 (0.8–1.21)
Gastroenterological Fraction	92	44 (48.3%)	34.6 ± 11.6	2.31 (1.63–2.99)	64.5 ± 11.3	1.07 (0.76–1.39)
Nephrological Fraction	213	48 (22.4%)	30.0 ± 13.2	1.42 (1.02–1.82)	51.4 ± 11.5	0.96 (0.68–1.23)
Sepsis	43	27 (62.8%)	32.7 ± 11.8	3.57 (2.22–4.91)	61.8 ± 11.6	1.58 (0.98–2.17)
Liver Failure^§^	50	31 (62.0%)	36.8 ± 11.9	2.59 (1.68–3.5)	68.3 ± 11.6	1.2 (0.78–1.62)
Chronic Heart Failure (NYHA IV)^§^	10	6 (60.0%)	38.5 ± 16.1	2.09 (0.42–3.77)	70.8 ± 9.8	1.06 (0.21–1.9)
Cirrhosis^§^	68	38 (55.9%)	36.2 ± 10.4	2.51 (1.71–3.3)	67.4 ± 10.2	1.12 (0.76–1.47)
Hemodialysis	70	29 (41.4%)	35.7 ± 12.2	1.84 (1.17–2.51)	57.2 ± 11.6	1.26 (0.8–1.72)
AKI	97	40 (41.2%)	34.7 ± 12.7	1.95 (1.35–2.55)	58.1 ± 11.4	1.22 (0.84–1.6)
Nosocomial Infection^§^	26	10 (38.5%)	31.4 ± 13.6	2.13 (0.81–3.64)	61.0 ± 15.6	0.96 (0.36–1.55)
CKD	131	45 (34.6%)	34.7 ± 13.7	1.54 (1.09–2)	58.2 ± 12.8	0.99 (0.7–1.28)
Vigilance Disturbance^§^	26	9 (34.6%)	37.5 ± 14.3	1.35 (0.47–2.23)	60.8 ± 15.4	0.87 (0.3–1.45)
Cardiac Arrhythmias^§^	52	17 (32.7%)	35.7 ± 14.2	1.37 (0.72–2.02)	53.4 ± 12.5	1.2 (0.63–1.77)
Non-invasive Ventilation	17	5 (29.4%)	31.5 ± 10.5	1.85 (0.23–3.47)	53.5 ± 8.8	1.17 (0.14–2.19)
Vasoactive Drugs^§^	188	55 (29.3%)	32.8 ± 12.4	1.56 (1.15–1.94)	57.3 ± 12.1	0.9 (0.66–1.14)

Data given as the mean and standard deviation. SD = Standard Deviation; SMR = Standard Mortality Ratio; CI = Confidence Interval; in CKD = chronic kidney disease, AKI = acute kidney injury

^§^ = part of the SAPS 2 or SAPS 3 scoring system

**Fig 3:**
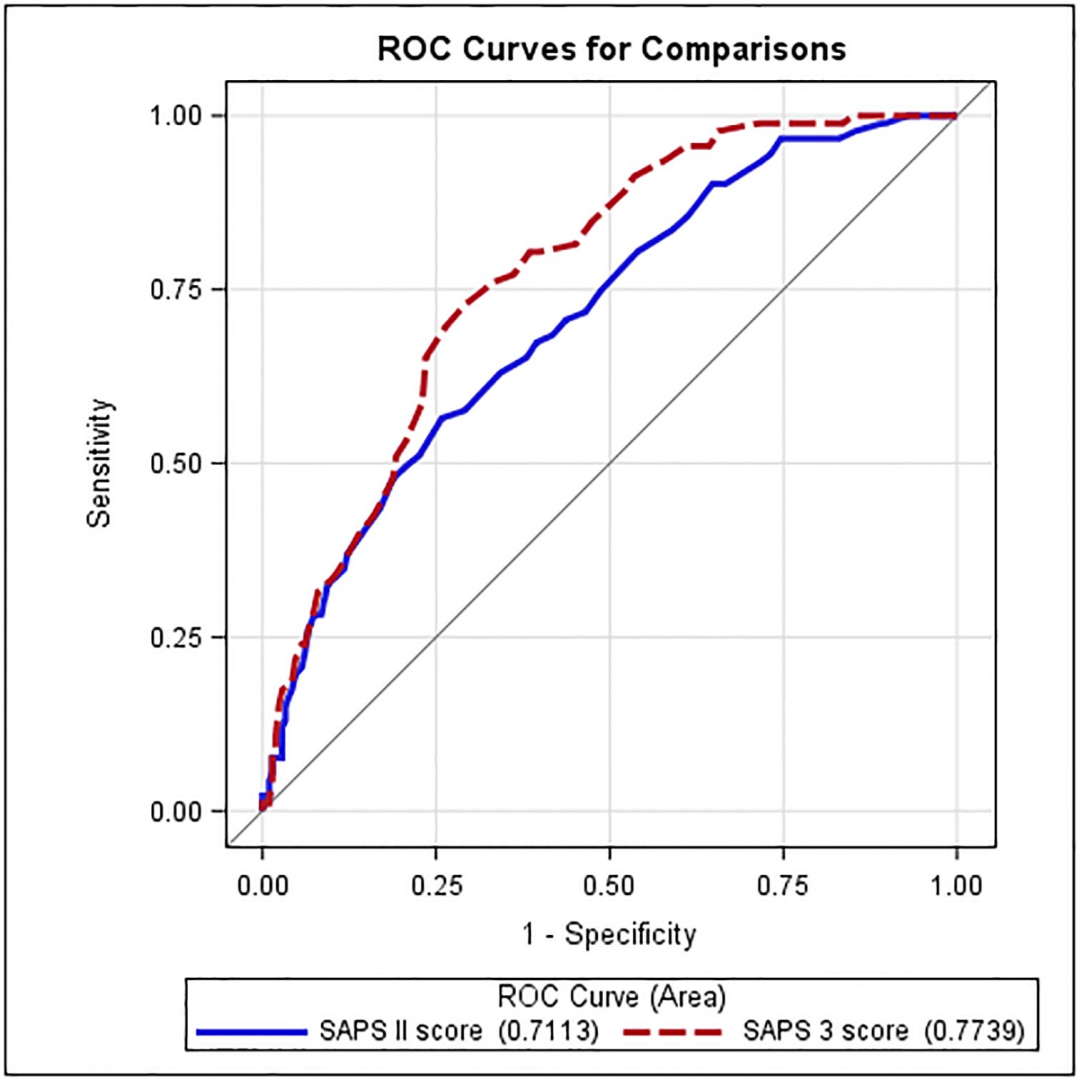
SAPS 2 and SAPS 3 discrimination performance. The SAPS 2 and the SAPS 3 showed acceptable discrimination performances, with an AUROC of 0.71 (95% CI, 0.65–0.77) and 0.77 (95% CI, 0.72–0.82), respectively, although there was no significant difference between both scores. Nevertheless, the SAPS 3 demonstrated superior SMR-based discrimination power compared to the SAPS 2.

With an AUROC of 0.71 (95% CI, 0.65–0.77) for SAPS 2 and 0.77 (95% CI, 0.72–0.82) for SAPS 3 (Table 4; Fig 3), both scores revealed an acceptable discrimination performance. However, calibration performance differed significantly. Based on the Calibration belt, SAPS 3 presented a good calibration (*P* = 0.906) while SAPS 2 showed a poor calibration, which was pronounced among patients with low risk of mortality (*P*<0.001, Table 4, Figs 1 and 2).

When viewed separately, the SAPS 2 and the SAPS 3 demonstrated an equally consistent performance in either nephrological or gastroenterological/hepatological patients. However, the SAPS 2 exhibited a poor performance in terms of SMRs in association with liver failure, cirrhosis and sepsis, which represented leading causes of mortality. In fact, the observed mortality for liver failure, cirrhosis and sepsis was 62.0%, 55.9%, and 62.8% respectively. Here, the SAPS 2 predicted mortality rates were 24.0% for liver failure, 22.3% for cirrhosis, and 17.6% for sepsis, resulting in a SMR of 2.59, 2.51, and 3.57, respectively (Table 5). Overall, the SAPS 3 demonstrated a more accurate SMR for each of these diagnoses along with a predicted mortality of 51.8% for liver failure, 50.0% for cirrhosis, and 39.8% for sepsis resulting in a SMR of 1.2, 1.12, and 1.58, respectively (Table 5).

## Discussion

In intermediate care patients, previous studies revealed an overestimation of mortality by either the SAPS 2 [18–20] or the SAPS 3 [18], which is largely consistent with analyses that evaluated these tools in critical care patients [6, 7, 14–16]. In the present work, we report a significant underestimation of mortality by the SAPS 2 in intermediate care patients with prolonged or end-stage liver and/or kidney diseases. To date, the SAPS 2 has often been suggested as most suitable tool for mortality prediction not only by earlier investigations in different ICU settings [14, 16] but also by a recent mixed population study conducted on a multi-purpose Spanish ImCU [18]. Contrary to these results, we found a slightly better discrimination performance along with a remarkably more accurate mortality prediction capacity for the SAPS 3 scoring system.

These conflicting results may be particularly attributable to the unique case mix of our study population. In contrast to three preexisting ImCU studies [18–20] or the original investigations that introduced the SAPS 2 and SAPS 3 scoring systems [26, 27], our study enrolled predominantly patients with chronic or terminal renal and hepatic diseases. Among those, transplant candidates or patients after SOT were represented in high percentages. The SAPS 3 comprises more variables linked to gastrointestinal and hepatic disorders (GHD), i.e., cirrhosis, liver failure, severe pancreatitis, acute abdomen, total bilirubin, platelets, and Glasgow Coma Scale (GCS), compared to the SAPS 2, which takes into account only three GHD-related parameters, i.e., total bilirubin, GCS, and sodium. Thus, it is not surprisingly that the SAPS 3 showed superior accuracy in mortality prediction for liver failure and cirrhosis than the SAPS 2, in our cohort. Even though Dupont et al. reported controversial findings in cirrhotic patients, our data are in line with a recent study by Alegre et al. that also attested superior test performance for the SAPS 3 compared to the SAPS 2 in patients with liver cirrhosis [21, 34]. Furthermore, differences between present study results and previous research are emphasized in septic patients; here, we observed the most pronounced underestimation of mortality risk using either score. Even though the SAPS 3 revealed better performance in terms of SMR relative to the SAPS 2, both scores were deemed unsuitable to adequately assess the risk of death among septic patients in the present cohort. However, due to the restricted sample size of this subgroup, this result should be considered with caution.

In terms of evaluating calibration performance, we consciously did not use the Hosmer-Lemeshow goodness-of-fit C-test [35]. Even though this test has been frequently used to validate mortality risk prediction scores [5–7, 10–12, 14, 15, 17, 18, 20, 26, 27], it has also been repeatedly criticized for different limitations related to its interpretation. For example, the Hosmer-Lemeshow goodness-of-fit C-test describes only deviations between observed and expected mortality, but neither contains information about their direction or dimension nor identifies characteristics of subgroups or individual patients that cause such deviations [36, 37]. Furthermore, high *P*-values do not necessarily provide evidence for a good fit, as numerous conditions can generate high *P*-values, including poor test power [36, 38]. In contrast to the Hosmer-Lemeshow goodness-of-fit C-test, the Calibration belt relates the logits of predicted and observed mortality in a polynomial function, whereby confidence intervals can be computed and plotted as the eponymous Calibration belt [29, 30]. The graphic illustration can assess the goodness of fit without any supervised categorization. Moreover, directions and ranges of poor calibrations from the perfect fit of a model can be directly visualized. The Calibration belt has already been tested against the Hosmer-Lemeshow goodness-of-fit C-test with similar type I error rates [30] and has been previously used in a similar study context [16]. For comparison purposes, we still applied the Hosmer-Lemeshow goodness-of-fit C-test and attached these results as supporting information (S1 and S2 Figs) below. In fact, using this method, we calculated an acceptable calibration performance for both prediction models.

Clearly, we agree with Lucena et al. that differences in case mix exert a strong influence on calibration and discrimination performance of the SAPS 2 and the SAPS 3 [18]. This consideration may gain additional support from our previous work that detected superior performance for the SAPS 2 relative to the SAPS 3 in ICU patients with internistic (predominantly cardiovascular) disorders at the same university hospital [14]. Of note, recent research analyzing data from three ICUs with over 2500 patients concluded that scoring systems with more predictor variables (such as the SAPS 3 when compared to the SAPS 2) are likely to achieve better overall performance relative to those with fewer [13].

Finally yet importantly, several characteristics and limitations of the present research warrant special attention. First, both the relatively small sample size and the unique case mix of this retrospective single-center study may generate substantial differences in terms of reproducibility of the SAPS 2 and the SAPS 3 based assessments between present and previous cohorts [18, 26, 27]. Moreover, different admission and discharge criteria as well as non-uniform staff- and/or shift- work patterns might skew or strongly particularize our findings, thus rendering them likely not extrapolable to other ImCU populations and settings. Just in this respect, it deserves attention that our ImCU had a less favorable nurse-to-patient ratio compared with the study conducted by Lucena et al [18], i.e., 1:4 vs. 1:3, respectively. In view of almost equal scores for the SAPS 2 (31.4 ± 12.9 vs. 36.6 ± 11.9) and the SAPS 3 (55.4 ± 12.9 vs. 58.4 ± 15.4), this condition may have been at least co-responsible for higher in-hospital mortality rates (30.2% vs. 20.1%) reported in the present study compared with the study by Lucena et al [18].

## Conclusions

The SAPS 3 (North Europe Logit) provided good discrimination and satisfactory calibration and was highly accurate in predicting mortality in our cohort of adults admitted to the ImCU due to chronic or terminal renal or gastrointestinal/hepatic disorders. The SAPS 2 appeared less suitable for risk evaluation in this setting due to a marked underestimation of mortality in comparison to the SAPS 3. Despite a large number of preexisting validation studies for SAPS 2 and SAPS 3 in ICU patients and (to a lesser degree also in) ImCU patients, predictive performance of these tools remains inconsistent and seems to be strongly influenced by variable case mix indices and other specific characteristics of the attending wards [10, 14, 18, 26, 27]. Furthermore, scoring systems are prone to be outdated over time as ImCU populations and structures change; this fate is driven by the evolution of diagnostic, therapeutic, technological, and economic means or strategies, which influence treatment outcomes and mortality. Thus, the present findings may not be easily generalizable to other populations. Especially, the poor performance of the SAPS 2 for sepsis and acute and/or chronic liver disorders seen in our analysis stresses the need for further and broader validation of these and other predictive ICU scores in different ImCU settings and populations during larger, prospective and well-designed studies.

## Supporting information

S1 Fig Calibration curve based in the Hosmer and Lemeshow goodness-of-fit C test for SAPS 2. Calibration performance for SAPS 2 (χ2 = 3.08; *P* = 0.876). Note that a high p value (>0.05) indicates a well-calibrated model. Calibration curves were created by plotting the predicted mortality (x-axis) against observed mortality (y-axis).
(TIF)

S2 Fig Calibration curve based in the Hosmer and Lemeshow goodness-of-fit C test for SAPS 3. Calibration performance for SAPS 3 (χ2 = 11.09; *P* = 0.196). Note that a high p value (>0.05) indicates a well-calibrated model. Calibration curves were created by plotting the predicted mortality (x-axis) against observed mortality (y-axis).
(TIF)
