# Supplementary figures and images for: The predictive performance of SAPS 2 and SAPS 3 in an intermediate care unit for internal medicine at a German university transplant center; A retrospective analysis
Source: PLoS One. 2019 Sep 25;14(9):e0222164. doi: 10.1371/journal.pone.0222164 (PMC6760764; doi:10.1371/journal.pone.0222164)

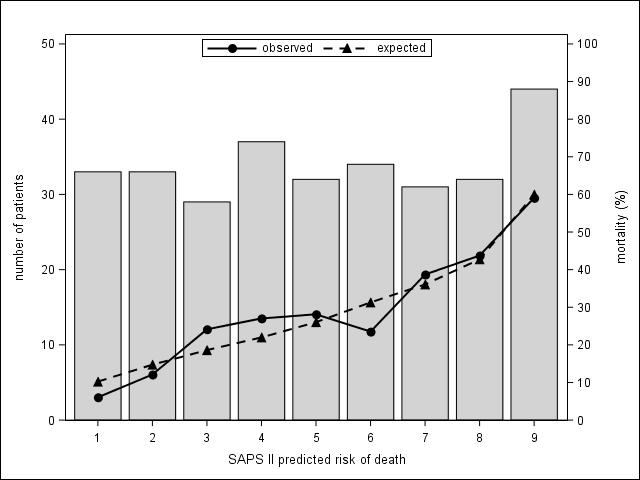

Supplement: S1 Fig — Calibration performance for SAPS 2 (χ2 = 3.08; P = 0.876). Note that a high p value (>0.05) indicates a well-calibrated model. Calibration curves were created by plotting the predicted mortality (x-axis) against observed mortality (y-axis). (TIF) [file pone.0222164.s001.tif]

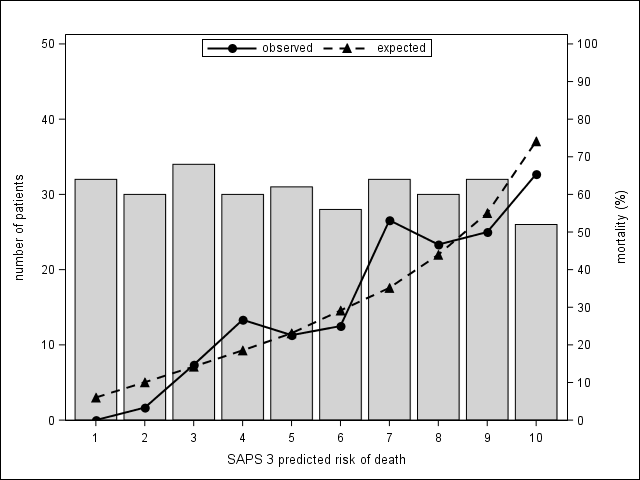

Supplement: S2 Fig — Calibration performance for SAPS 3 (χ2 = 11.09; P = 0.196). Note that a high p value (>0.05) indicates a well-calibrated model. Calibration curves were created by plotting the predicted mortality (x-axis) against observed mortality (y-axis). (TIF) [file pone.0222164.s002.tif]
